# Multifunctional biodegradable polyvinyl alcohol/ nanolignin composite films of optical, photoluminescence, antimicrobial properties and enhanced fruit preservation ability

**DOI:** 10.1038/s41598-025-24553-z

**Published:** 2025-11-10

**Authors:** Mona Abdelrehim, Fawzy G. El Desouky, Maysa E. Mohram

**Affiliations:** 1 Packaging Materials Department, Chemical Industries Research institute, National Research Centre, Dokki, Giza Egypt; 2 Solid State Physics Department, Physics Research Institute, National Research Centre, Cairo, Giza 12622 Egypt; 3 Microbial Chemistry Department, Biotechnology Research Institute, National Research Centre, 33 El Behooth St., Dokki, Giza Egypt

**Keywords:** Nanolignin, Optical properties, Antimicrobial, Active packaging, Fruit preservation, Chemistry, Materials science, Nanoscience and technology

## Abstract

Lignin is the second most abundant natural polymer after cellulose. It is an affordable renewable material with versatile properties that enable its utilization in many technological applications. This work is concerned with the preparation of nanocomposite biodegradable films based on nanolignin (NL) and polyvinyl alcohol (PVA). Different ratios of NL ranging from 0.5% to 8% were added to the PVA solutions, and free-standing films are obtained by casting technique. The formed films were characterized using FTIR and XRD and their morphology was studied using high- resolution scanning electron microscope (HRSEM). Studying the optical properties of the films revealed that the films have a high UV-shielding ability that is boosted with an increasing ratio of NL in the film. Moreover, the presence of NL reduced the optical band gap of polyvinyl alcohol. The concentration of NL determines the equilibrium between PL enhancements and quenching, demonstrating a concentration-sensitive interaction between the lignin-conjugated electrons and the PVA matrix. Antimicrobial activities toward different microorganisms are boosted by increasing the amount of NL in the prepared films. The films demonstrated obvious antioxidant properties (radical scavenging activity, RSA%), and films containing 8% NL showed 82% RSA. Moreover, gas barrier properties and the ability of the prepared formulations to preserve plum fruit in terms of weight and color are examined. The obtained nanocomposite films demonstrated versatile properties that enable variable applications, including active packaging material.

## Introduction

Lignin is regarded as the second-largest renewable source after cellulose. It is an aromatic compound and the most abundant polymer to obtain carbon^1^. It contributes to the cross-linking of cellulose and forms around 30% of woody plant tissues. It offers cell division hardness, strength, and inelasticity. Since lignin is insoluble in water, intentionally twisted, and devoid of hydrolyzable connections, it is an extremely complicated molecule for enzymatic depolarization^2^. Yearly, the paper and pulp industries produce roughly 50 million tons of lignin worldwide, of which about 2% is recovered for use in the production of chemicals^3,4^. Over the past decade, lignin has seen a sharp rise in interest from the scientific and business communities due to the increased awareness of climate change and the urgent need to reduce industrial pollution^5^. Lignin has been extensively researched and utilized as an antioxidant, UV-resistant, and antibacterial additive besides being regarded as a carbon precursor and biobased filler in biomedical engineering^6–8^. In its nanoscale form, lignin has found extensive application as a reactive filler in a range of thermoplastic and thermosetting polymers, including phenolic^9^, epoxy resin^10,11^, polyethylene^12^, poly(lactic acid)^13^, poly(methyl methacrylate)^14^, polyurethane^15^. Nanolignin (NL) played the primary role as a UV-blocking agent in the majority of these systems alongside other bioinspired nanofillers exclusively studied for the effective development of UV shielding properties of polymeric materials^16^.

Polyvinyl alcohol (PVA), a semi-crystalline and hydrophilic polymer, has received particular attention because of its exceptional organic biocompatibility and solvent resistance. PVA is widely utilized in food packaging, medical treatments, and building materials^17^. Nevertheless, PVA has poor biological activity, limited resistance to moisture absorption, and weak thermal stability as an active packaging material^18^. PVA is frequently combined with other biopolymers or biomass-derived nanofillers, such as nanocellulose^19^, chitin/chitosan, or starch to enhance its qualities and functions^20,21^. PVA with NL added has already been shown to exhibit improved mechanical and thermal characteristics as well as UV-blocking, antioxidant, and antibacterial capabilities^22^. The incorporation of lignosulfonic acid in PVA matrix yielded a high-toughness nanocomposite and a breaking strain value of 282% which was attributed to dense hydrogen bonding between PVA and lignosulfonic acid^23^. Cross-linked films of PVA alkali lignin were reported by Su et al.^24^ The films crosslinked by glutaraldehyde and plasticized using glycerin revealed satisfying mechanical and thermal properties at ratios of PVA and alkali lignin of 5:1 and 1.67% 7.1% (w/w) for Glutaraldehyde and glycerin respectively. Recently, chemically cross-linked films of PVA incorporated with lignin nanoparticles were thoroughly explored by Yang et al.^25^ Two different crosslinkers of varied functionality were used, citric acid and glutaraldehyde. The effect of crosslinker molar ratio and amount of filler on thermal stability and mechanical properties of the formed films was investigated. The addressed works used crosslinkers to achieve free standing films focusing on studying the effect of the films as active packaging material. In this study, NL extracted from Bagasse is incroporated in PVA matrix in different ratios to prepared nanocomposite films without additives as crosslinkers or plasticizers with the aim of preparing edible coating suitable for fruit preservation. The influence of adding different ratios of the nanofiller on optical, mechanical, gas barrier properties as well as antimicrobial, and antioxidant activities, was investigated. Furthermore, optical properties and Photoluminescence (PL) analysis of the nanocomposite films are studied.

## Materials and methods

### Materials

Lignin sodium sulfonate extracted from Bagasse was prepared in the labs of the paper and cellulose department-National Research Centre after the method described in the literature^26^. Polyvinyl alcohol (degree of polymerization 1700–1800) was purchased from Qualikems Fine Chemicals Pvt. Ltd., India. Tetrahydrofuran (THF) 99.9% was a product of Sigma and used as received.

### Methods

#### Synthesis of nanolignin (NL)

The method of NL is described before^27^ with some modification, typically: 2 g of lignin sulfonate is dissolved in 200 mL of tetrahydrofuran (THF) and stirred well till complete dissolution. 160 mL of distilled water is added at rate of 4 mL/min as a non-solvent. After removal of THF by rotary evaporation, the formed NL powder is separated by centrifuge at 5000 rpm and finely dried and maintained for characterization.

#### Synthesis of PVA film

Polyvinyl alcohol (PVA) solution was prepared by dissolving 10 g of the polymer in 100 mL of hot distilled water. The solution was stirred well until complete dissolution of the polymer then poured in a glass dish of suitable size, and dried in the oven at 70 °C.

#### Synthesis of nanocomposite films (PVA/NL)

For preparing (PVA/NL) nanocomposite the polymer solution was loaded with different ratios of NL, namely 0.5, 1, 3, 5, and 8% with respect to the amount of PVA. The casted films were dried overnight at 70 °C.

#### Antimicrobial properties testing

The antibacterial activities were carried out in the Microbial Chemistry Department, National Research Centre, using the diffusion plate method. A piece of each film was placed on a plate (9 cm diameter) containing a solid bacterial medium (nutrient agar), which has been seeded with the spore suspension of the test organism. After incubation at 37 °C for 24 h, the diameter of the clear zone of inhibition surrounding the tested film is taken as a measure of the inhibitory power of the sample against the particular test organism. The antimicrobial activity of the tested samples was examined with gram-positive bacteria, *Staphylococcus aureus* ATCC 6538, and gram-negative bacteria *Escherichia coli* NRRN 3008, and pathogenic yeast *Candida albicans* EMCC 105. The obtained results are compared with the reference antibiotic that was purchased from Egyptian markets.

#### Studying of antioxidant properties

To determine radical scavenging activity (RSA), specimens of each film (0.26 g) were put in Falcon tubes containing 3 ml of methanol and shaken for 1 min. The tubes were kept at ambient temperature for 2 h before analysis. A methanolic solution of DPPH (3 mL of 0.04 mM) was added and shaken vigorously. The solutions were incubated for 30, 60, 90, and 120 min at room temperature in the dark, and then UV absorbance at 517 nm was measured. As a control, the same procedure was performed but without any film samples. The DPPH radical scavenging activity was calculated using Eq. (1).

1 $${\text{RSA }}\% {\text{ }} = {\text{ }}\left[ {\left( {{\text{A}}_{{{\text{Control}}}} {-}{\text{A}}_{{{\text{Sample}}}} } \right)/{\text{ A}}_{{{\text{Control}}}} } \right]{\text{ }} \times {\text{ 1}}00$$

#### Investigation of moisture retention ability

Film composites were tested for their water retention capability by cutting film samples of similar dimensions (1 × 3 cm) and weighing them (W_b_). The films are dried in the oven at 60 °C for 6 h then weighed again (W_a_). The moisture retention capability was determined using the following formula:

2 $${\text{Moisture Retention Capability }}\left( \% \right) = ~~w_{b} - {\text{ }}w_{a} /w_{b} \times {\text{ 1}}00$$

#### Testing of fruit preservation

Solutions of PVA/NL formulations were prepared in a concentration of 10 w%. The plum fruits purchased from the local market were washed thoroughly and dried. The initial weight of the fruit was determined (*wi*). Then each fruit was dipped in the PVA/NL solution for 10s. Finally, the coated fruits were left at room temperature, and the visual changes were observed with time. The coated fruits were weighed after 15 and 20 days.

### Characterization

#### Particle size determination

Investigation of the size of the NL particles was performed using particle size analyzer- Nano-ZS. Malvern Instruments Ltd., UK.

#### FTIR

The chemical structure of the prepared materials was studied with a Bruker VERTEX 80 ATR-FTIR instrument (Germany) combined with Platinum Diamond ATR, comprising a diamond disk. The instrument has internal reflector measure spectra in the range of 400–4000 cm^− 1^ with a resolution of 4 cm^− 1^ and a refractive index of 2.4.

#### XRD

The structure of the produced samples was investigated using powder X-ray diffraction (PXRD) technique and a diffractometer from Empyrean Panalytical Instruments in the Netherlands, with CuK as an X-ray source of radiation.

#### Surface morphology investigations

High resolution transmission electron microscope (HRTEM) JEOL Model, JEM-2100, Japan, was used to examine the microstructure of the samples.

#### Optical properties

The optical diffuse reflection spectra of the samples were recorded at ambient temperature using an optical spectrophotometer (Jasco model V-570) across a wavelength range of 190–2500 nm. The photoluminescence (PL) spectra were obtained using an FS5 spectrofluorometer produced by Eidenburg, UK.

#### Investigation of mechanical properties

**Fig. 1:**
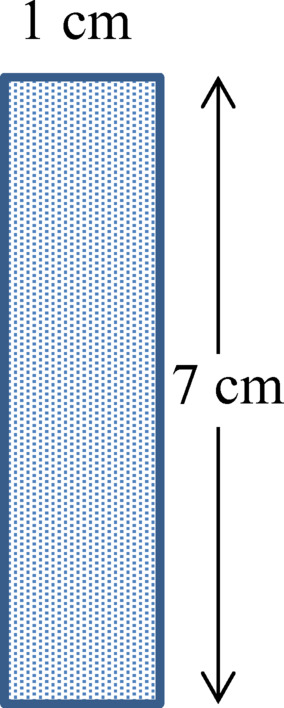
Shape and dimensions of film sample used for mechanical testing.

Mechanical properties of the film samples were measured using instrument from INSTRON- 34SC-5. Samples were cut in strips (1 cm width X 7 cm length) and three different strips were measured for each sample. Figure 1 The sample was placed in the device and crosshead speed 20 mm/min was used. The mean value and standard deviation was reported.

#### Gas permeability testing

Gas permeability investigations were carried out using N530-B Gas Permeability Analyzer from GBPT Packaging Equipment Co.

## Results and discussion

**Fig. 2:**
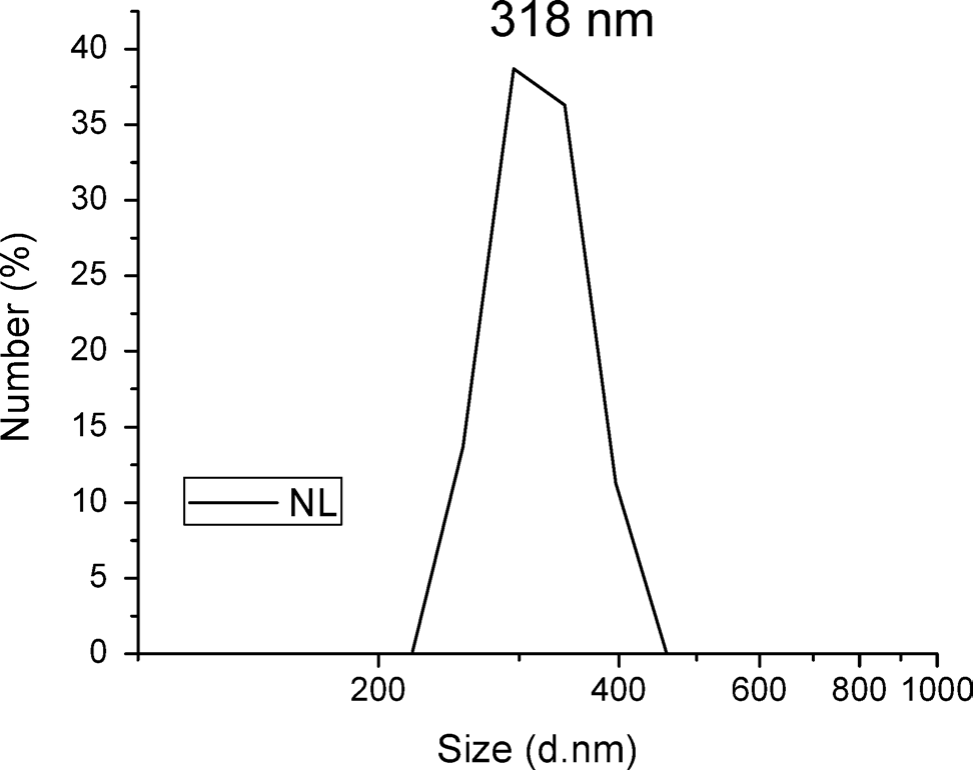
Distribution of NL particle size.

**Fig. 3:**
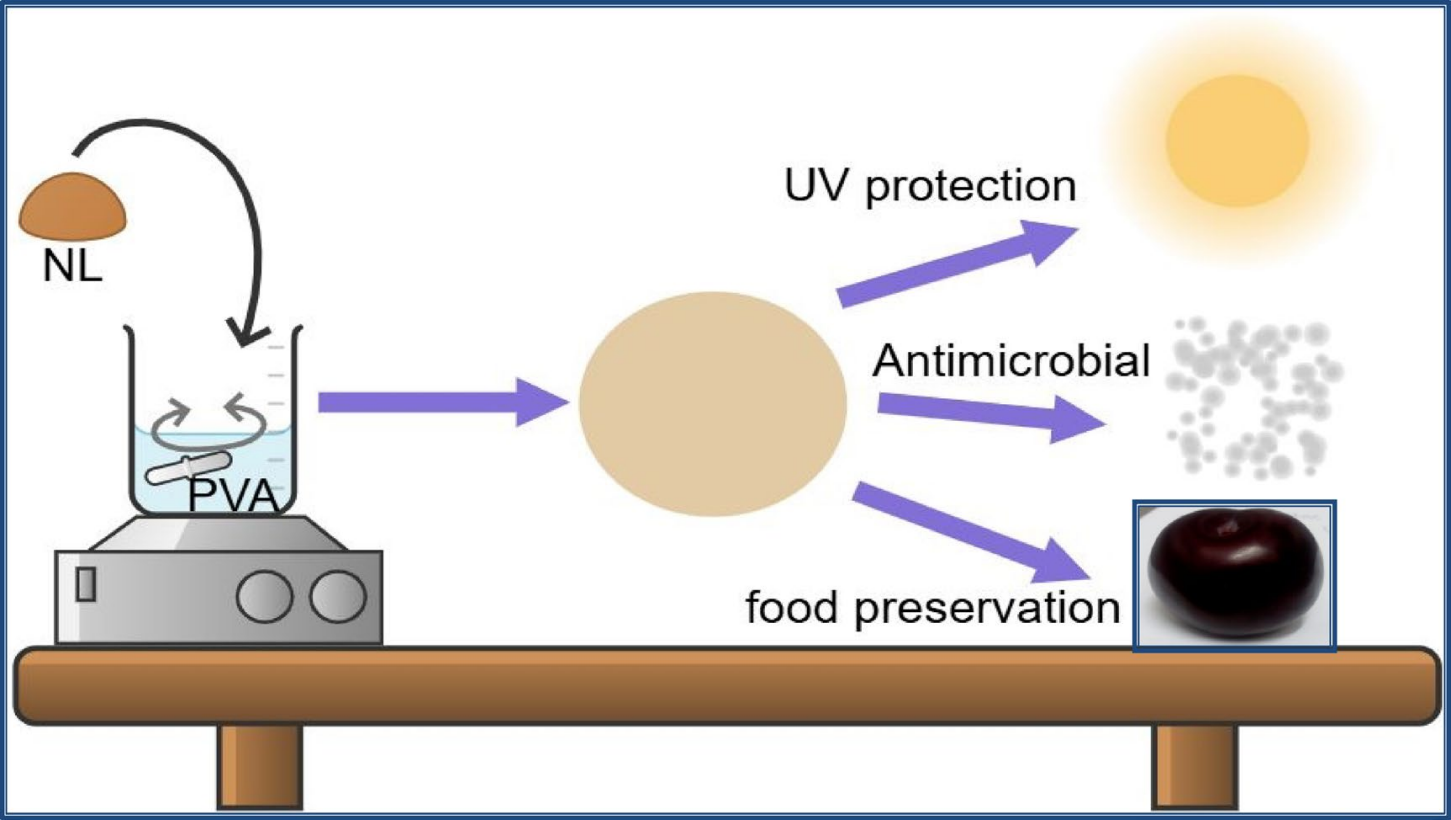
Schematic representation for preparation of PVA/NL composites for different applications.

Investigation of NL particle size was carried out using Zeta-sizer. The results showed that the average particle size of the prepared NL is 318. (Fig. 2) It should be pointed out that the size of NL is affected with different parameters among them the rate of water dropping, as fast water addition leads to formation of particles of smaller diameters^28^. The obtained NL was used as a nanofiller to PVA as a multifunctional nanocomposit. Free standing films were prepared for testing in different applications as illustrated in Fig. 3.

### FTIR

**Fig. 4:**
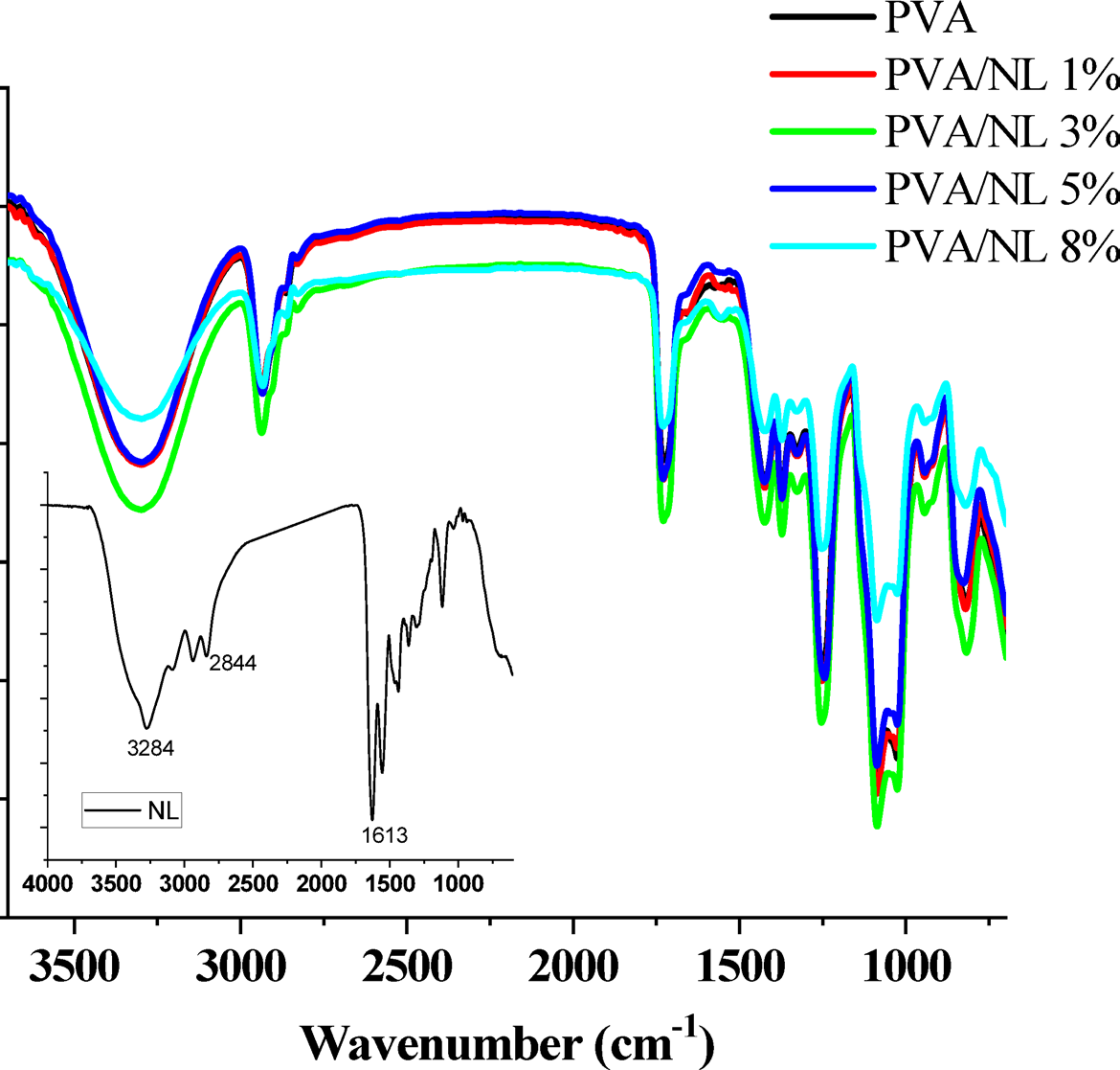
FTIR spectra of PVA/NL nanocomposites (inset is FTIR of NL).

Chemical structures of nanocomposite films of different ratios of NL have been investigated using FTIR. The spectra depicted in Fig. 4 revealed the following bands: OH stretching vibration at 3294 cm^− 1^, aliphatic C-H stretching at 2943 and 2855 cm^− 1^. A band at 1733 cm^− 1^ corresponding to C = O group and C-H deformation appears at 1428 cm^− 1^^29^. The chemical structure of lignin contains many functional groups such as hydroxyl, carbonyl, aromatic rings and methoxy moieties^30^. The FTIR spectrum of NL shown as an inset in the figure depicted that the band of OH group can be found at 3284 cm^− 1^, band of C-H stretching appeared at 2844 cm^− 1^ while that band at 1613 cm^− 1^ can be attributed to C = O group^31^. The change in band positions between NL (spectrum shown in the inset) and that of the PVA/NL suggested the formation of hydrogen bonding between functional groups in PVA and NL.

### XRD

**Fig. 5:**
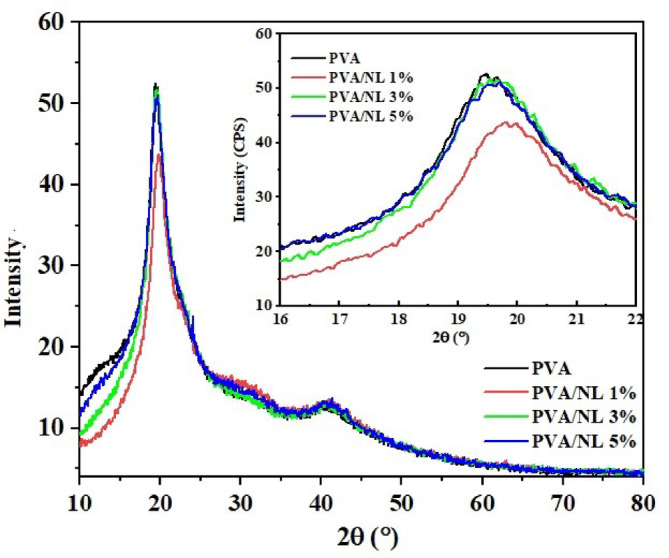
XRD pattern of PVA and the prepared nanocomposites.

The nanocomposite films are investigated by XRD and the obtained diffraction patterns are shown in Fig. 5. PVA is a semi-crystalline polymer with a characteristic diffraction peak at 2θ = 19.5^o^ which corresponds to (101) structural plane^32^. This peak depicts also inter- and intramolecular hydrogen bonding. Upon the addition of various ratios of NL, the crystallinity of PVA was found to exhibit a slight change, as demonstrated in Fig. 3 as an inset. Yang et al. pointed out that Yang et al. pointed out that the crystallinity peak of PVA diminishes with increasing NL concentration can be attributed to disrupting PVA ordering by the crosslinker, as evidenced by the emergence of a new peak at 2θ = 15°^25^. Here, even with the addition of NL to the polymer matrix, the main peak positions of PVA are slightly shifted. By applying the Scherrer formula to the samples of PVA, PVA/NL 1%, PVA/NL 3%, and PVA/NL 5%, the diameters of the crystallites were found to be 5.2 nm, 5.4 nm, 5.3 nm, and 5.5 nm, respectively.

### SEM

**Fig. 6:**
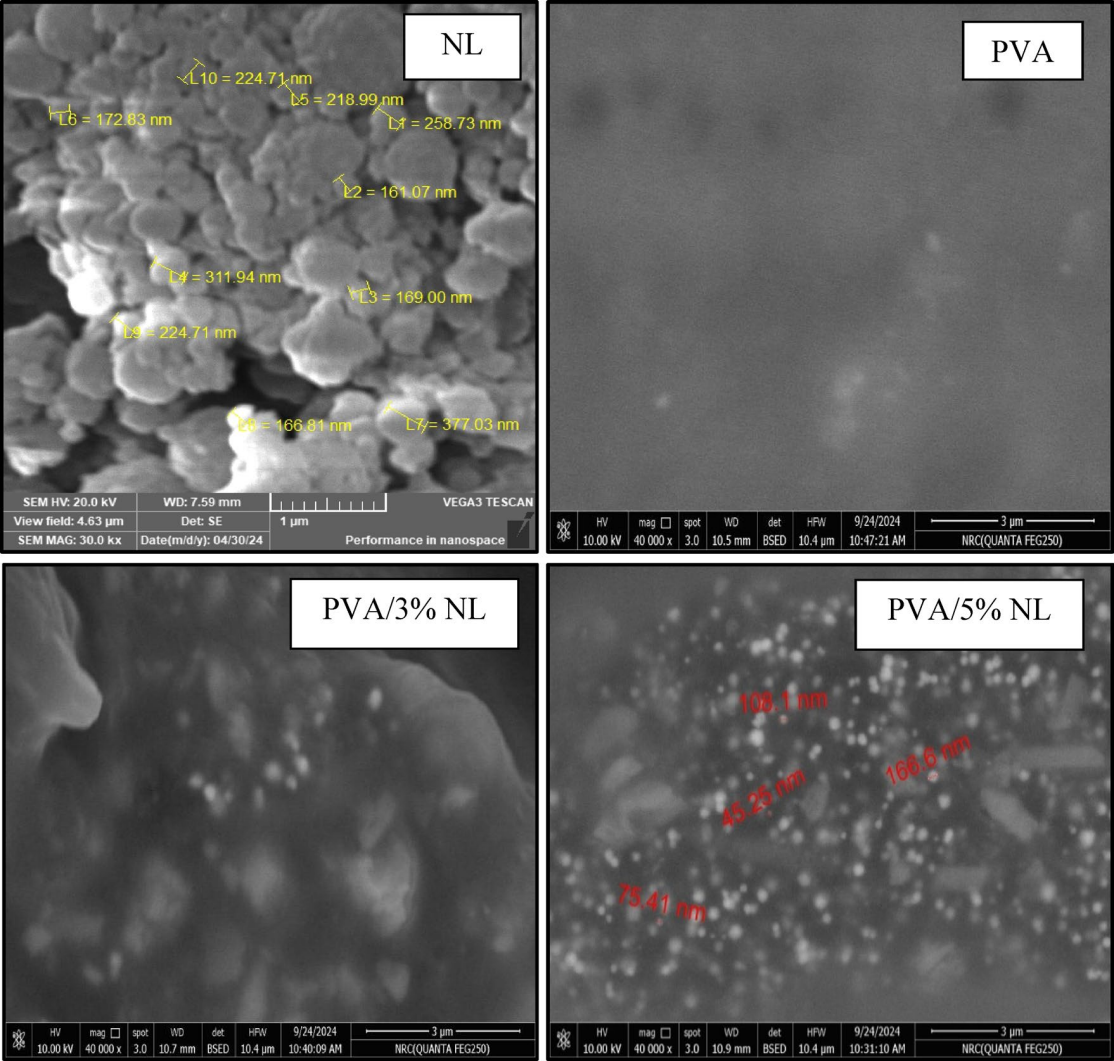
Scanning electron microscope images of PVA, PVA/ 3%NL and PVA/5%NL films (X = 40 K).

The prepared NL has a spherical structure as revealed by SEM of diameter ranges from 166 to 378 nm. Figure 6 However, morphology investigation of film samples PVA, PVA/3% NL, and PVA/5% NL revealed a smooth surface in the case of pure PVA (figure 4) while nanolignin spherical shape appeared in the composite films PVA/3% NL and PVA/5% NL. Nevertheless, since the amount of NL is relatively high, aggregates of the filler could be assigned in the sample PVA/5% NL and their diameter was found to be in values between 45 and 166 nm. (Figure 6) The reduction in NL size in the film sample can be attributed to high shear due to stirring while preparing the film solution.

### Optical properties

**Fig. 7:**
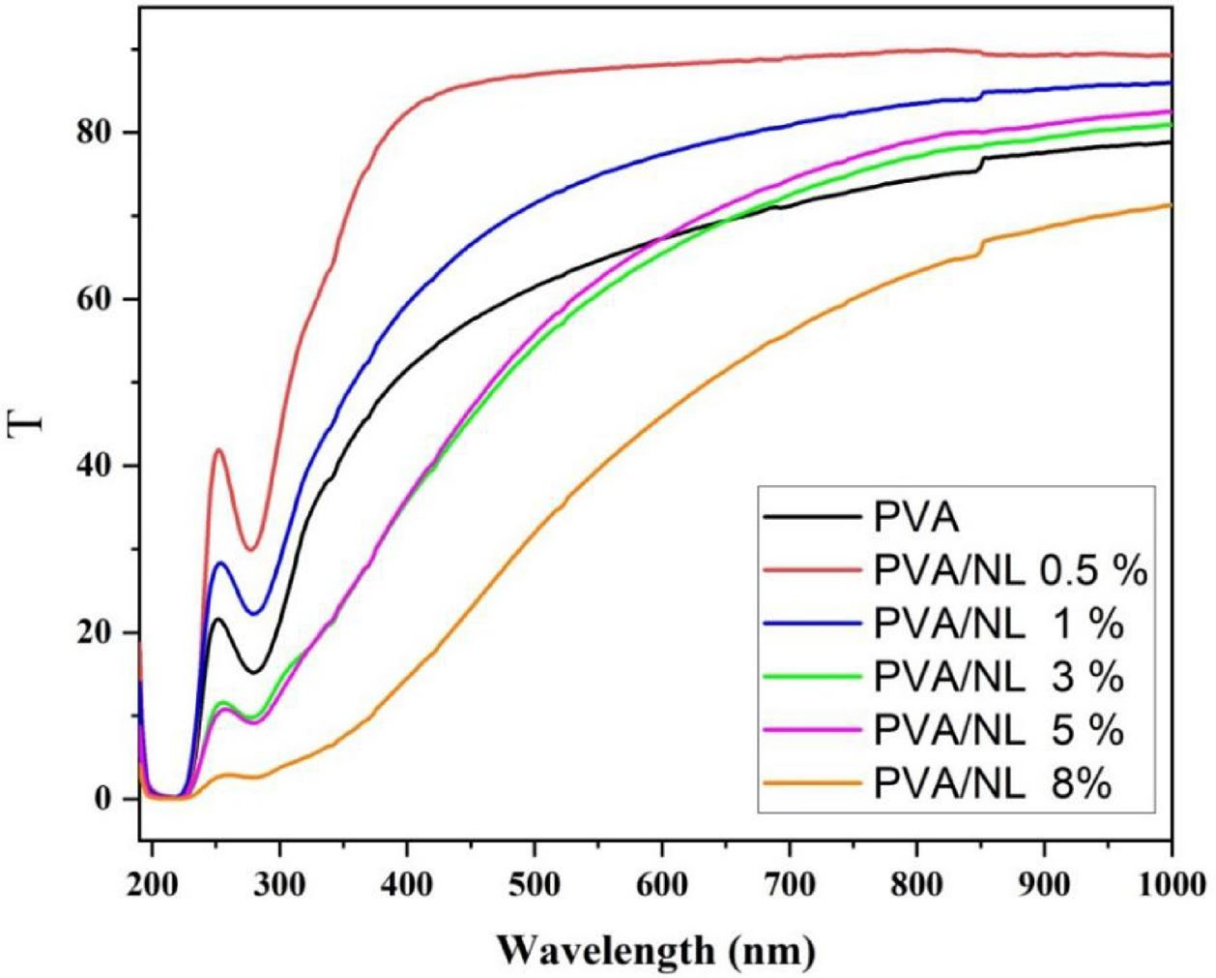
Transmissions vs. wavelength plot for polyvinyl alcohol (PVA) films incorporated with various concentrations of nanolignin.

Figure 7 shows a transmission vs. wavelength curve for films made of polyvinyl alcohol (PVA) that has different amounts of NL. The x-axis indicates the wavelength in nanometers (nm), which runs from 200 nm to 1000 nm and encompasses the UV, Vis, and NIR regions. The diagram depicts the impact of NL incorporation on the optical properties of PVA films. As the quantity of NL rises, UV transmission diminishes, suggesting that NL may contribute to UV radiation attenuation, hence enhancing the material’s UV-protective properties. Nanolignin (like PVA-NL 8%) in higher concentrations makes light more likely to be absorbed or scattered, as shown by a drop in transmission. The changes in transmission are caused by the interactions between NL particles and the PVA matrix. These interactions may change the material’s optical properties and its ability to be used as protective films or coatings. So, as NL concentration increases, the transmission decreases especially below 400 nm, suggesting an enhanced ability of the films to absorb or scatter UV light^33^.

The increase in transmission at particular wavelengths may occur if the nanolignin content is slight (0.5%, 1% NL) or if its dispersion within the PVA matrix is non-uniform. PVA (Polyvinyl Alcohol) is a water-soluble synthetic polymer with outstanding film-forming, adhesion, and emulsifying capabilities. The PVA transmittance of about 60% was recorded before^34^ Moreover, many factors affect optical properties of PVA films such as crosslinking due to inter and intrahydrogen bonding, polymer molecular weight, and polymer concentration as revealed by C. Ding et al.^35^ PVA has high hydrogen bonding due to the multiple hydroxyl (OH) groups along its chain. Nanolignin is a complex polymer found in plant cell walls that adds stiffness. Nanolignin is lignin at the nanoscale. Although it hydrophobic, it contains polar functional groups can form hydrogen bonds with PPVA. When nanolignin is introduced at low concentrations, it can effectively position itself between PVA chains. The hydroxyl groups on the nanolignin can compete with the PVA chains for hydrogen bonding to happen. Nanolignin particles may be finely spread out at 0.5% and 1% NL, which stops them from sticking together and scattering yielding nanocomposite of higher transparency.

**Fig. 8:**
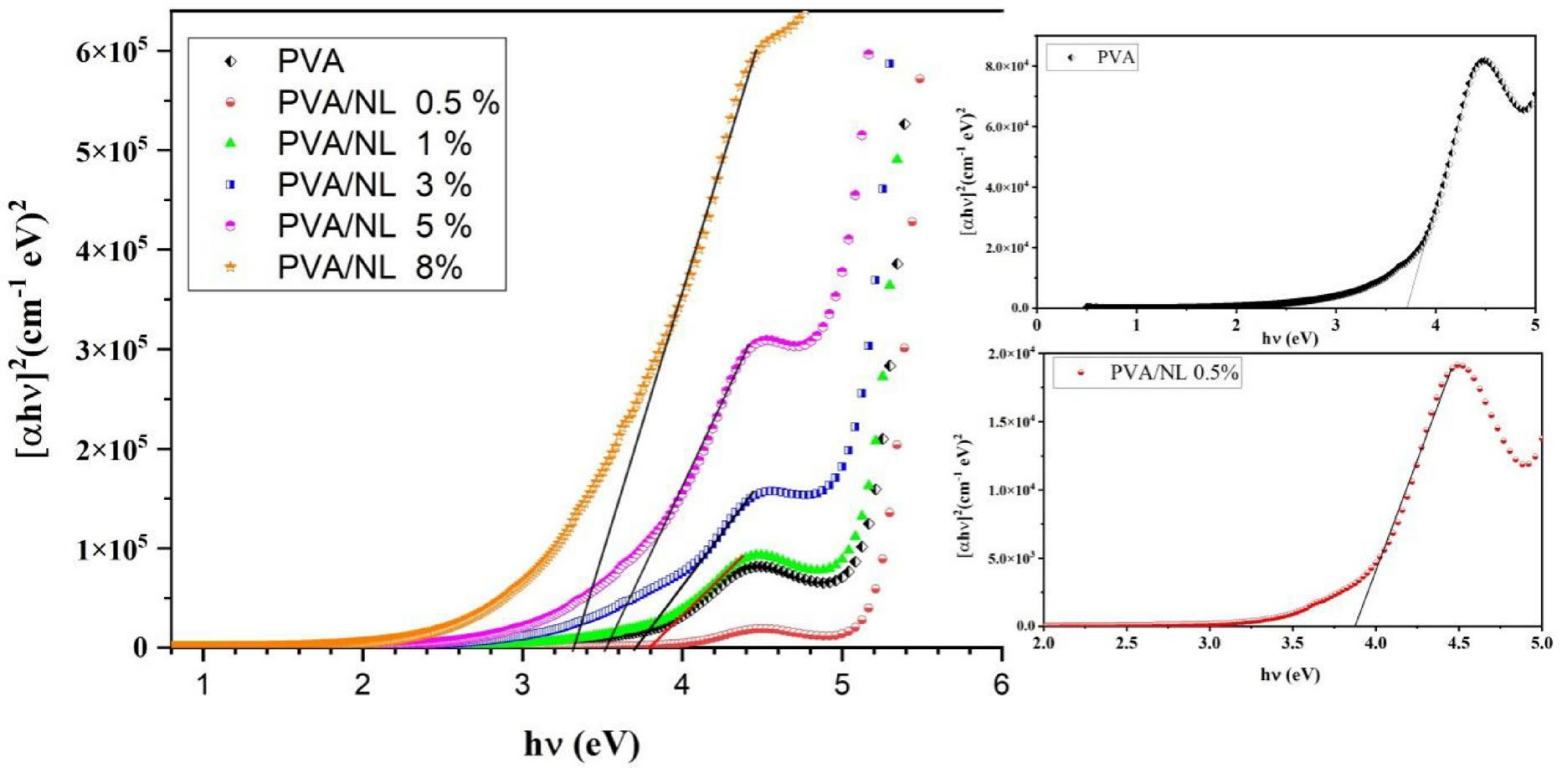
Tauc plot illustrating the effect of different concentrations of nanolignin on the direct band gap of a polyvinyl alcohol (PVA) polymer.

The optical properties of films that contain both NL and PVA are controlled by the interactions between the PVA matrix and the nanolignin particles. Examining the following mechanisms can help us understand these links. Lignin, a complex polymer present in plant cell walls, exhibits strong light-absorbing properties, especially in the ultraviolet (UV) spectrum (200–400 nm). Nanolignin particles can absorb a substantial amount of ultraviolet (UV) light due to their diminutive size and extensive surface area. The decrease in light transmission through the coating is due to this absorption. The reduction in transmission rate, which correlates with an increase in nanolignin content, demonstrates this. Furthermore, Lignin, a complex polymer present in plant cell walls, possesses a significant ability to absorb light, especially within the ultraviolet (UV) spectrum (200–400 nm). It is possible for nanolignin particles to absorb a large amount of UV light because of their small size and enormous surface area. This results in a reduction in the amount of light that passes through the film. An increase in NL concentration leads to a proportional decrease in transmission, thereby, illustrating this phenomenon^36^. The second advantage of NL particles is their heterogeneity, which allows them to scatter light. Light can scatter when it interacts with particles or structures that have a wavelength equivalent to the incident light on them. The scattering effects become increasingly pronounced with higher concentrations of NL, ultimately leading to a reduction in overall transmission. Third thing to think about is that NL is hydrophobic, but polyvinyl alcohol (PVA) is a hydrophilic polymer. Nanolignin can interact with PVA through hydrogen bonding, van der Waals forces, and maybe even π-π interactions. The presence of nanolignin may enhance the optical density of the material, thereby decreasing transmission levels, which improves opacity because of its inherent light-absorbing besides scattering properties, particularly at shorter wavelengths^37,38^. The phrase “optical transmission” refers to the process of determining optical absorption coefficients and band gap energies by employing a segment of an incoming electromagnetic field, as illustrated in Fig. 8 by Eq. (3)^38–40^.

3 $$\:\alpha\:\left(h\vartheta\:\right)=\frac{1}{d}\text{ln}\left[\frac{1}{T}\right]\:\:\:\:\:\:\:\:\:\:\:\:\:\:\left(3\right)$$

Where $\:\alpha\:\:is\:the\:$absorption coefficients, h (blank constant),$\:\:\vartheta\:\:\left(the\:frequency\right)$, d (thickness ), and T (transmittance).

This plot shows how varying quantities of nanolignin can tune the optical band gap of PVA. These alterations could be advantageous for applications that require particular optical features. (Fig. 8) illustrates a Tauc plot showing the effect of NL on the direct band gap of polyvinyl alcohol (PVA). In the presence of a rise in the concentration of NL, the curves display a leftward shift, which indicates a decrease in the energy of the optical band gap. This drop is particularly noticeable at higher concentrations, where it decreases from 3.7 eV to 3.3 eV. This trend shows that NL can lower band gaps in polymers by creating localized states and improving the manner of the electrons interaction within the polymer matrix. The introduction of nanolignin accomplishes this. Research has demonstrated that the aromatic structure of lignin enhances absorption in composites based on PVA, positioning it as a potential component for environmentally friendly and functional materials^35^. The incorporation of NL reduces the optical band gap of polyvinyl alcohol (PVA), potentially enhancing the material’s capacity to absorb photons with lower energies. This, in turn, increases the material’s value for applications in optoelectronics and solar energy harvesting^40,41^.

### Photoluminescence (PL) analysis

**Fig. 9:**
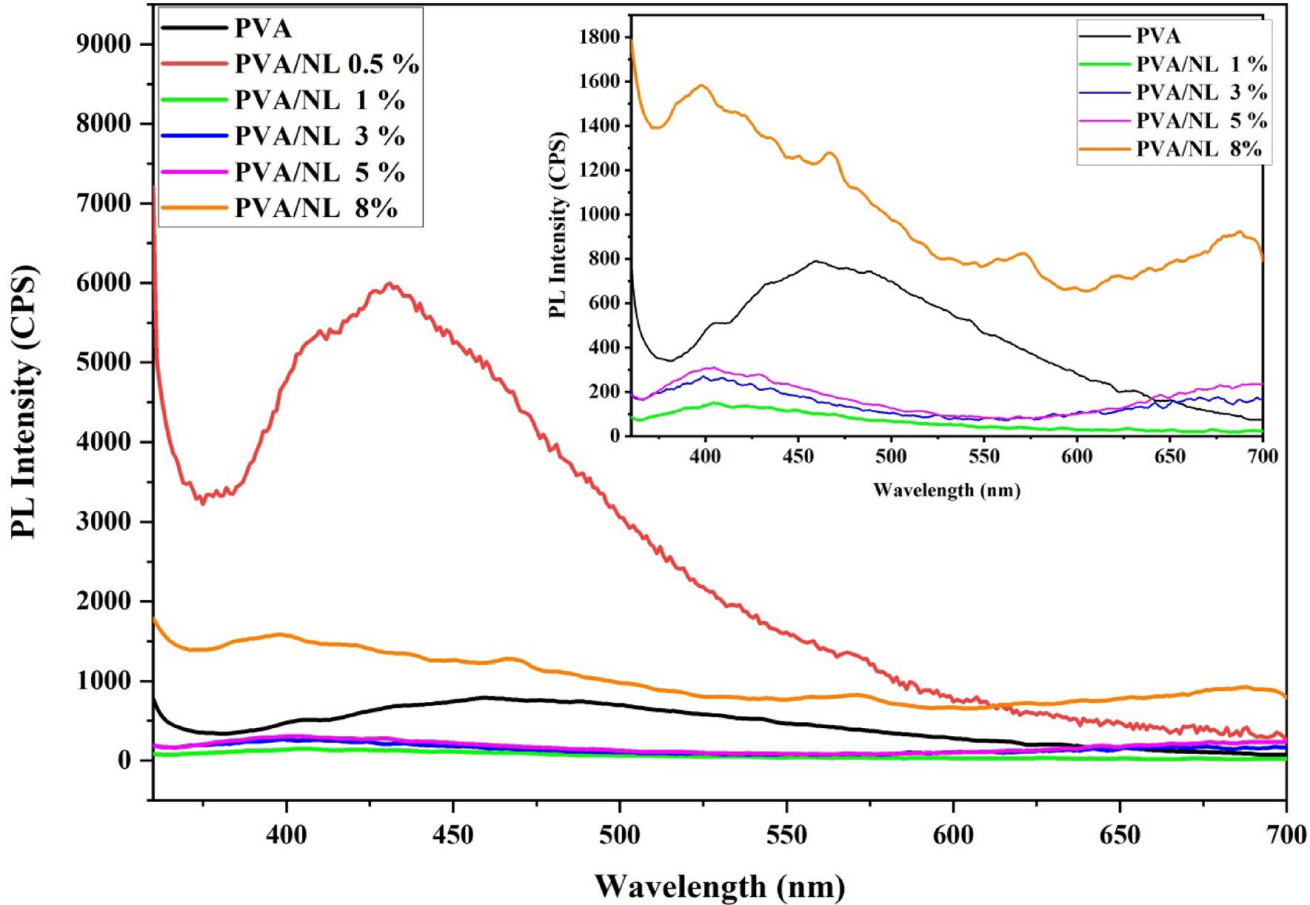
PL spectra of different concentrations of NL in the polyvinyl alcohol (PVA) matrix (inset is magnified spectra).

Through the utilization of photoluminescence (PL) techniques and methods, it is possible to conduct an examination of the molecular composition of a variety of substances, which may include the identification of structural faults or impurities^42^. An explanation for the PL behavior of polyvinyl alcohol (PVA) with nanolignin, which is depicted in the image, can be found in the interaction between the polymer matrix of PVA and the nanolignin particles, which affects the mechanisms that transmit energy at the excitation wavelength 350 nm. PVA molecules glow in the visible range (400–550 nm) because of the $\:{\pi\:}^{*}\to\:n$ electronic transition in free $\:-OH$ groups, which depends on how they are arranged inside oriented PVA molecules^43,44^. Our experiments revealed abroad mission peak for the pure PVA at 460 nm, and two shoulder peaks at 435 nm and 489 nm as shown in Fig. 9 as an inset.

Figure 9 depicts the photoluminescence (PL) spectra of polyvinyl alcohol (PVA) at various nanolignin concentrations. PVA in its purest form displays a baseline PL response; however, the incorporation of NL alters the composition of the PL features. Additionally, the sample that contained 0.5% nanolignin had the highest PL intensity, particularly between 400 and 550 nm, which indicated improved emission peaks at 410 nm, 426 nm, and 431 nm. This was most likely because nanolignin induced higher electronic transitions throughout the sample. Because of its aromatic nature, nanolignin not only acts as an energy donor at concentrations of 0.5% nanolignin, but it also makes it easier for electrons to flow between molecules. The aromatic rings within the lignin molecule consist of $\:\pi\:$ -conjugated systems.

These systems interact with polyvinyl alcohol (PVA) to generate new localized states within the band gap. By creating new ways for excitation and radiative recombination, these states improve photoluminescence (PL), which ultimately leads to PL becoming brighter in the 400–550 nm range^36^. The PL intensity, on the other hand, decreases at higher concentrations (for example, 1,3 and 5% NL), most likely as a result of a phenomenon known as “aggregation-induced quenching”. When NL particles come together, they reduce the amount of surface area that is accessible and block excitation sites. This results in an interruption of electron transit and a reduction in the efficiency of recombination. Furthermore, large concentrations of lignin can result in non-radiative recombination. These phenomena reduce the intensity of photoluminescence (PL) by causing excited electrons to lose energy in the form of heat rather than light. The concentration of nanolignin determines the equilibrium between PL enhancement and quenching, demonstrating a concentration-sensitive interaction between the lignin-conjugated electrons and the PVA matrix^36^.

**Fig. 10:**
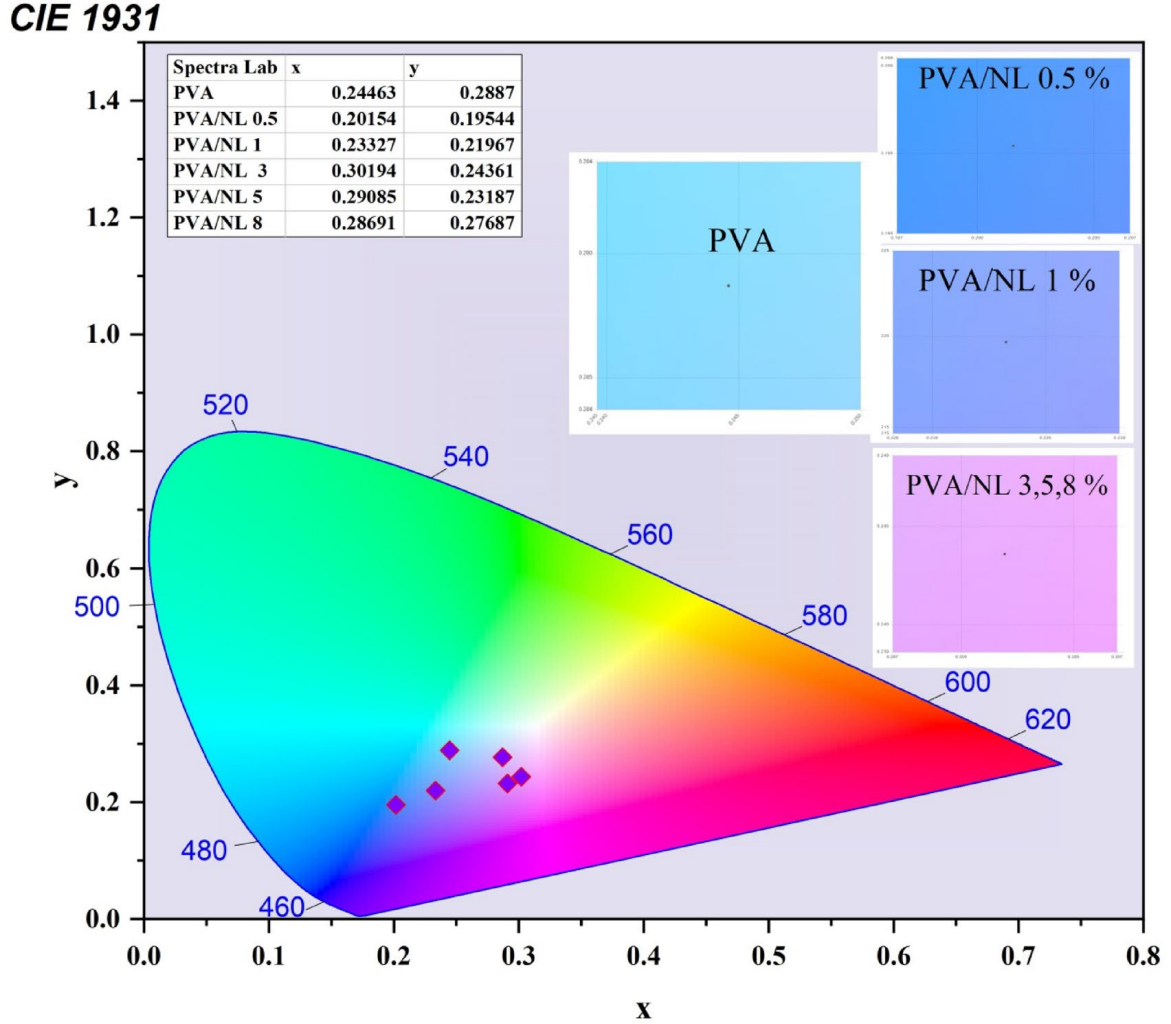
The International Commission on Illumination (CIE) for the effect of different concentrations of NL on polyvinyl alcohol polymer.

Figure 10 displays a representation of the diagram that the International Commission on Illumination (CIE) developed^45^. This figure depicts the chromaticity map for several different electroluminescent spectral zones, 0.24463 and 0.2887 are the coordinates that the CIE uses to describe the blue emission of PVA. There are significant shifts in CIE coordinates that occur as a result of the influence of different concentrations of NL on polyvinyl alcohol (PVA) polymer. At a concentration of 0.5%, the polymer exhibits a bluish (deep sky blue) emission, transitioning to light blue at 1% and ultimately transforming into magenta at concentrations of 3%, 5%, and 8%.

### Water retention capability

**Fig. 11:**
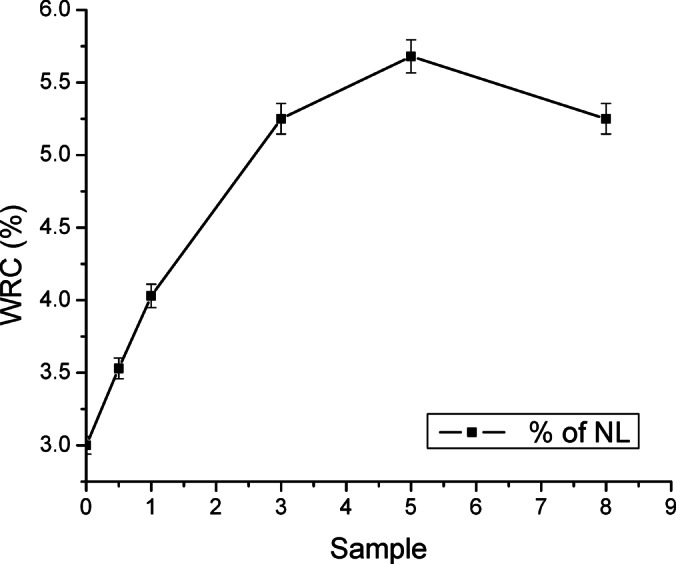
Water retention capability (WRC %) of tested samples.

The ability of the prepared film composites is studied, and the results shown in Fig. 11 depict that there is a gradual increase in water retention capability (WRC) by raising the amount of incorporated NL in the PVA matrix. This result can be attributed to the hydrophilic character of PVA and the presence of OH groups, which make hydrogen bonds with water molecules likely to occur^46^. However, the presence of pores or miniaturized size voids led to such an elevation in water uptake^47^. Also, the difference in WRC% values between samples PVA/NL 1% and PVA/NL 3% is higher than that between PVA/NL 1% and PVA/NL 0.5%. The highest WRC% was found to be 5.55% for sample PVA/NL 5%. The results also showed that the value of WRC% in the case of sample PVA/NL 8% started to lower again, giving the value of 5.2%, which can be explained by the presence of a high content of NL of hydrophobic nature affecting the ability of the nanocomposite to retain water^25^.

### Mechanical properties

**Fig. 12:**
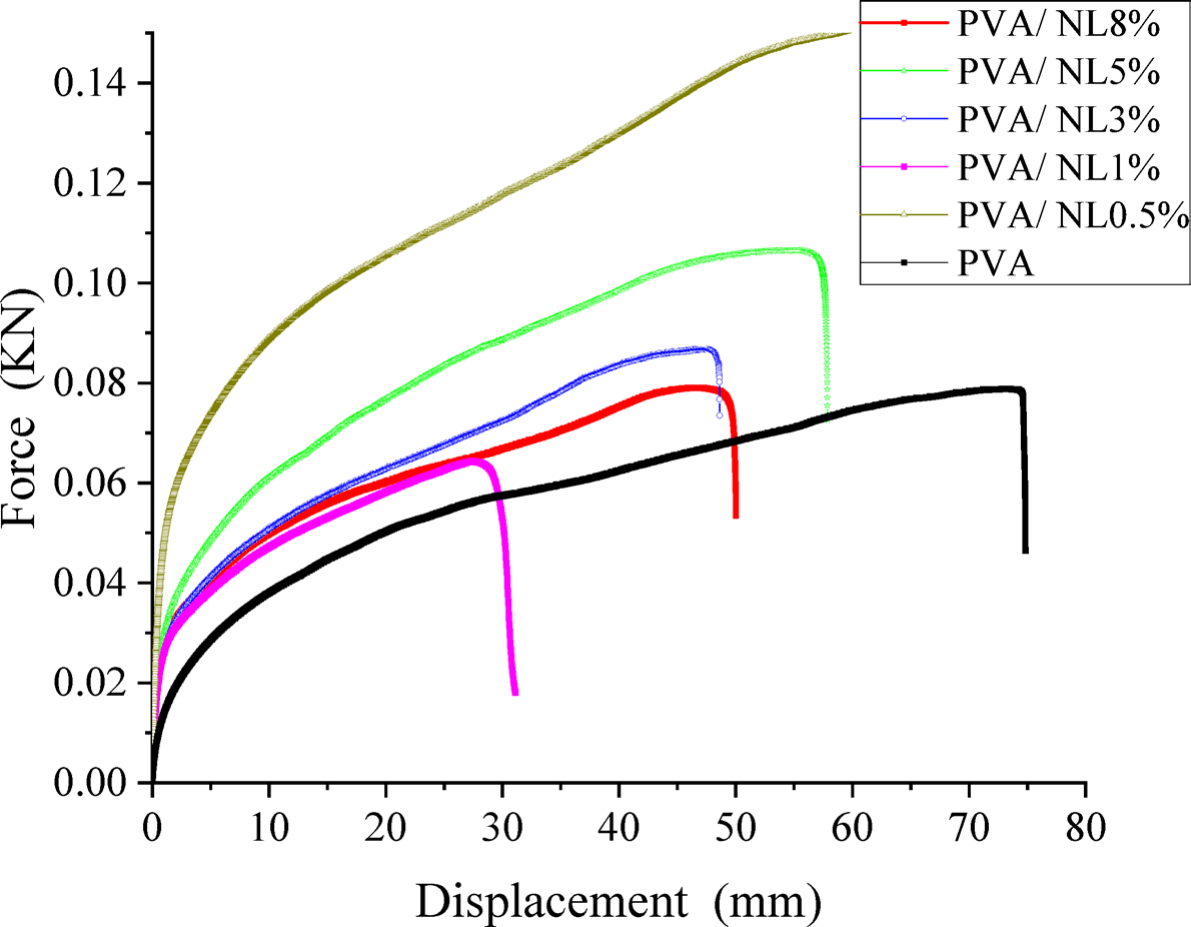
Force against displacement for PVA and its composites with nanolignin.

The effect of force on the PVA and its nanocomposite films is shown in Fig. 12. Generally speaking, all prepared films depicted good mechanical performance. It can be observed that PVA film has the highest displacement under applied force. However, the effect of NL addition varies according to its quantity in the nanocomposite film; for example, sample PVA/NL 0.5% showed the highest elongation at break % value of 328% (Table 1). But the composites samples revealed slight increase in tensile strength with increasing amounts of added NL. This behavior was observed on modifying PVA/ gelain blends with different ratios of Cinnamomum zeylanicum extract as repored by N. Gürler ett al.^48^ Moreover, Young’s modulus of nanocomposite films is obviously higher than neat PVA, reaching 4 folds for the sample containing 0.5% NL and more than 6 folds for sample PVA/NL 8%. These results can be attributed to the rigid nature of the lignin structure, which contains a large number of benzene rings^49^. The complexity of the stereostructure of the nanocomposite fosters the load transfer from PVA to NL and assists filler dispersion in the PVA matrix^50^. Rico-García et al. reported that uniform distribution of NL in the PVA matrix increased chain mobility, leading to high stretchability^51^. Nevertheless, the interfacial stress transfer due to hydrogen bonding between hydroxyl groups of PVA chains and polar groups in NL which was confirmed by the shift in the O-H stretching band (Fig. 4), This interaction restricted the chains’ mobility and increases the modulus.

**Table 1:** Tensile strength and elongation at break of prepared PVA/NL samples compared with pure PVA (*P* = ± 0.05).

Sample	Tensile strength (MPa)	S.D. (MPa)	Elongation at break (%)	Young’s Modulus (MPa)
PVA	33.45	4.9	249.0	134.7
PVA/NL 0.5%	35.8	1.6	328.0	580.9
PVA/NL 1%	33.0	3.1	110.4	710.8
PVA/NL 3%	41.0	4.6	181.0	521.3
PVA/NL 5%	38.6	2.7	173.7	483.9
PVA/NL 8%	30.5	1.0	166.0	707.7

### Radical scavenging activity

**Fig. 13:**
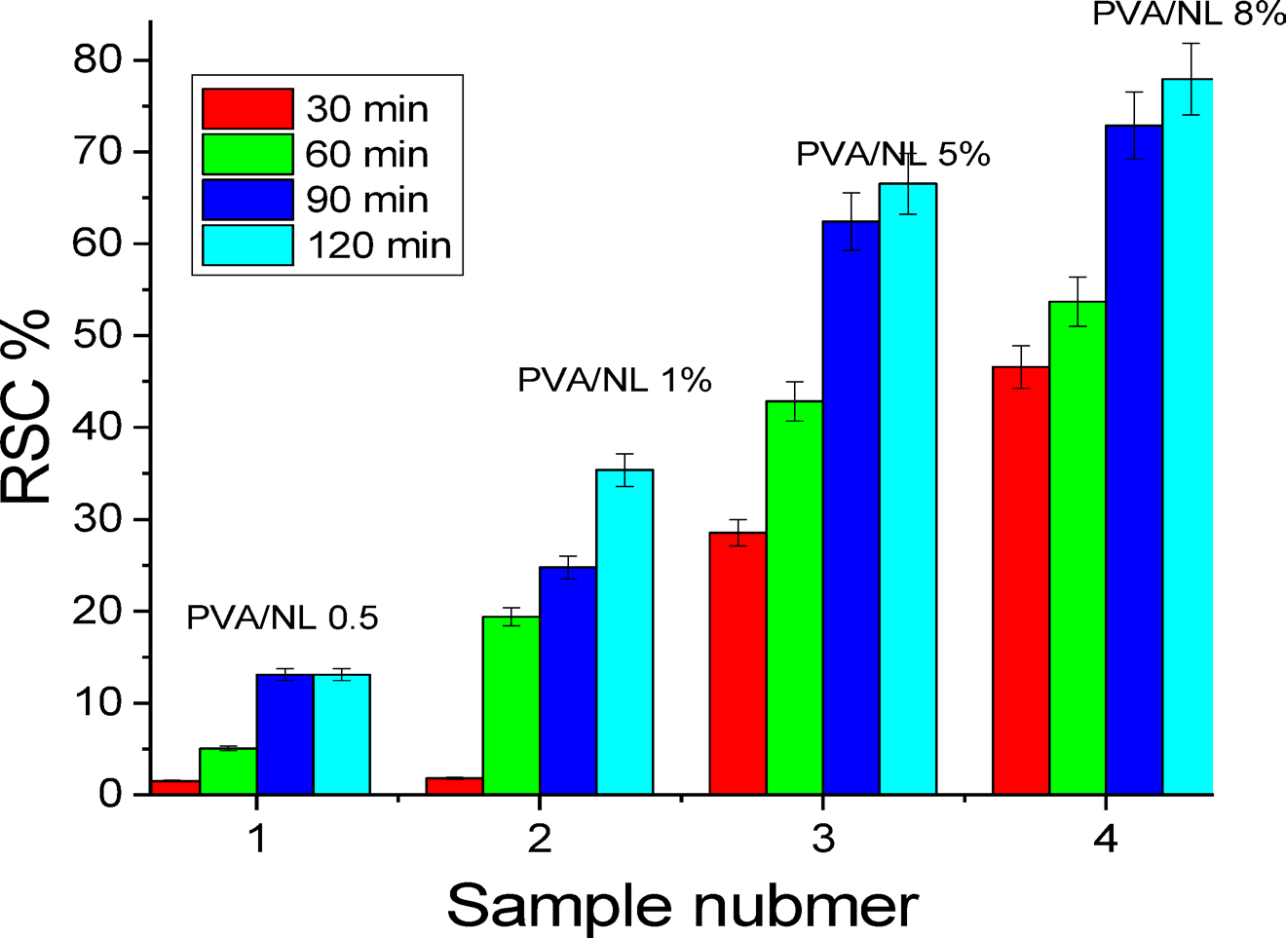
Radical scavenging activity (RSA%) of PVA/NL films of different composition (*p* < 0.05).

Oxidation of fats or lipids under the action of free radicals produced under the action of O_2_ causes food spoilage. Elimination of these free radicals is essential to protect food and extend its shelf life. Active packaging is a packaging material that contains additives with free radical removal capability. The antioxidant activity of the prepared PVA/NL films of different compositions was investigated. Polyvinyl alcohol has no antioxidant activity^25^, however, the value of RSA% = 20 after 90 min. upon the addition of only 0.5% NL to the PVA film. (Fig. 13) It was observed that values of RSA% are increased significantly by extending the test time from 30 to 120 min. Moreover, the highest RSA% value was for the sample PVA/NL 8%, which gave 78% after 120 min. These remarkably high antioxidant activities observed for tested film samples can be attributed to the presence of free phenolic groups of NL. It should be pointed out that PVA films containing crosslinked NL showed maximum RSA% values of 14%^25^.

### Gas permeability

Investigation of gas barrier properties for packaging films is crucial since O_2_ causes deterioration of backed food especially those that contain fats or lipids. Many factors affect gas permeability through packaging film among them; filler loading ratio, adhesion between filler and polymer matrix, filler shape, dispersion level, porosity, polarity of the material and immobilization of polymer chains^52^. The values of the gas permeability study shown in Table 2 revealed that barrier properties of the prepared films decreased by increasing amount of added NL, the lowest value was 12.56 g/m^2^.day for sample PVA/NL 1%. This can be explained by the good dispersion of NL in PVA matrix created tortuous paths and the diffusion path length of the gas in the film is increased^53^. However, nanocomposite films containing 3, 5, and 8% NL showed increasing tendency. This result can be attributed to the presence of filler aggregates in the film composite leading to facilitating gas mobility through the film^54,55^.

**Table 2:** O_2_ gas permeability values for different nanocomposites films.

PVA	PVA/NL 0.5%	PVA/NL 1%	PVA/NL 3%	PVA/NL 5%	PVA/NL 8%
38.91	31.95	12.54	21.31	42.70	87.9

### Antimicrobial activity

**Fig. 14:**
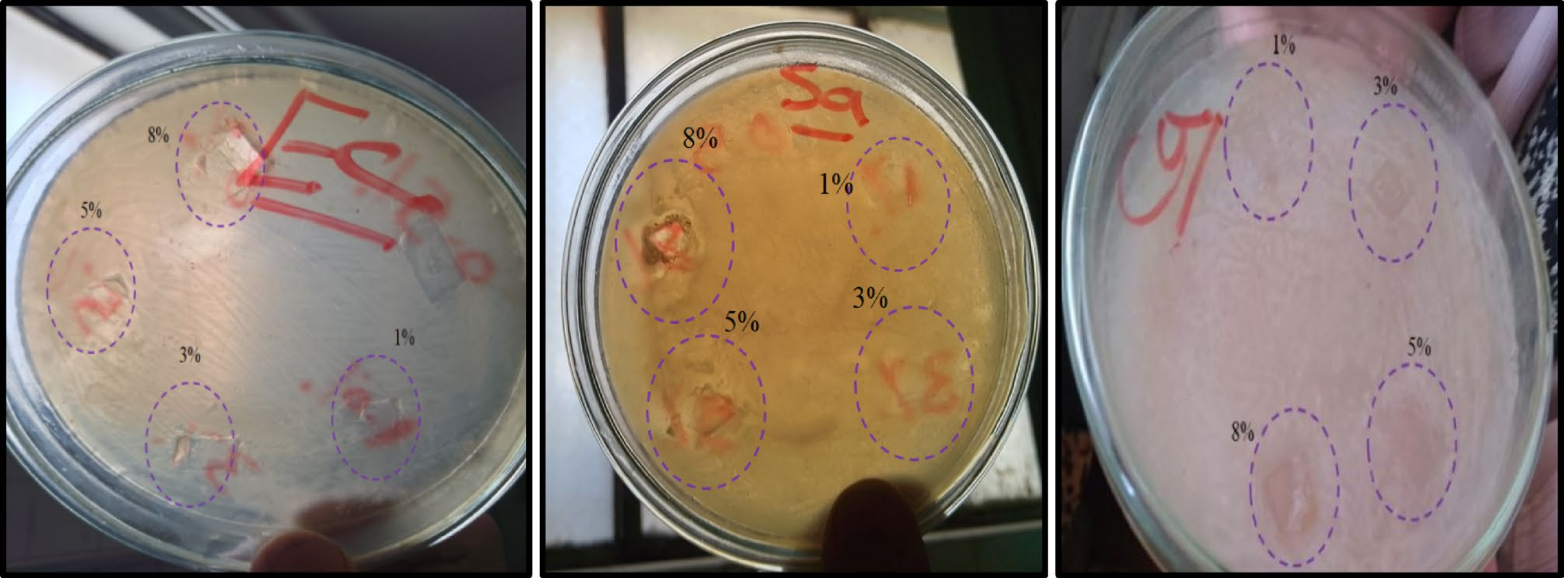
Images of clear inhibition zones of various microorganisms on using nano-composite films in different concentrations.

Foodborne microorganisms associated with food spoilage are one of the main concerns that should be restrained. Thus antimicrobial effectiveness is an essential feature for packaging film. Antimicrobial activity of the prepared nanocomposite films has been studied toward *Escherichia coli (E. coli)*,* Staphylococcus aureus (S. aureus)*, and *Candida albicans*. The prepared films demonstrated altered antimicrobial responses according to the nature of the microorganism and the amount of NL in the nanocomposite films as shown in Table 3 and photos. (Fig. 14) The antimicrobial behavior against *S. aureus* and *Candida albicans* increased clearly by increasing the amount of NL in the tested film sample although the values of inhibition zone diameter were lower than that of reference antibiotic. However, the films demonstrated antibacterial activity against *E. coli* nearly as the reference, especially the sample PVA/NL1%. These results can be explained in the light of presence of oxygenated functional groups in the NL structure and many hydroxyl end groups. It was reported that these functional groups can produce reactive oxygen species (ROS) such as superoxides or hydroxyl radicals^56,57^. These ROS groups can attack the microorganism cell membrane causing the release of the cell contents and consequently its death^58^.

**Table 3:** Antimicrobial activity of the PVA/NL composite films.

Microorganism	Inhibition zone (mm)						
	Conc. of NL						
	0	0.5	1	3	5	8	Reference Anti- biotic
*Escherichia coli*	9	14	15	15	15	13	15
*Staphylococcus aureus*	11	13	15	16	18	20	30
*Candida albicans*	12	15	16	17	20	27	40

### Composite films preservation capability

**Fig. 15:**
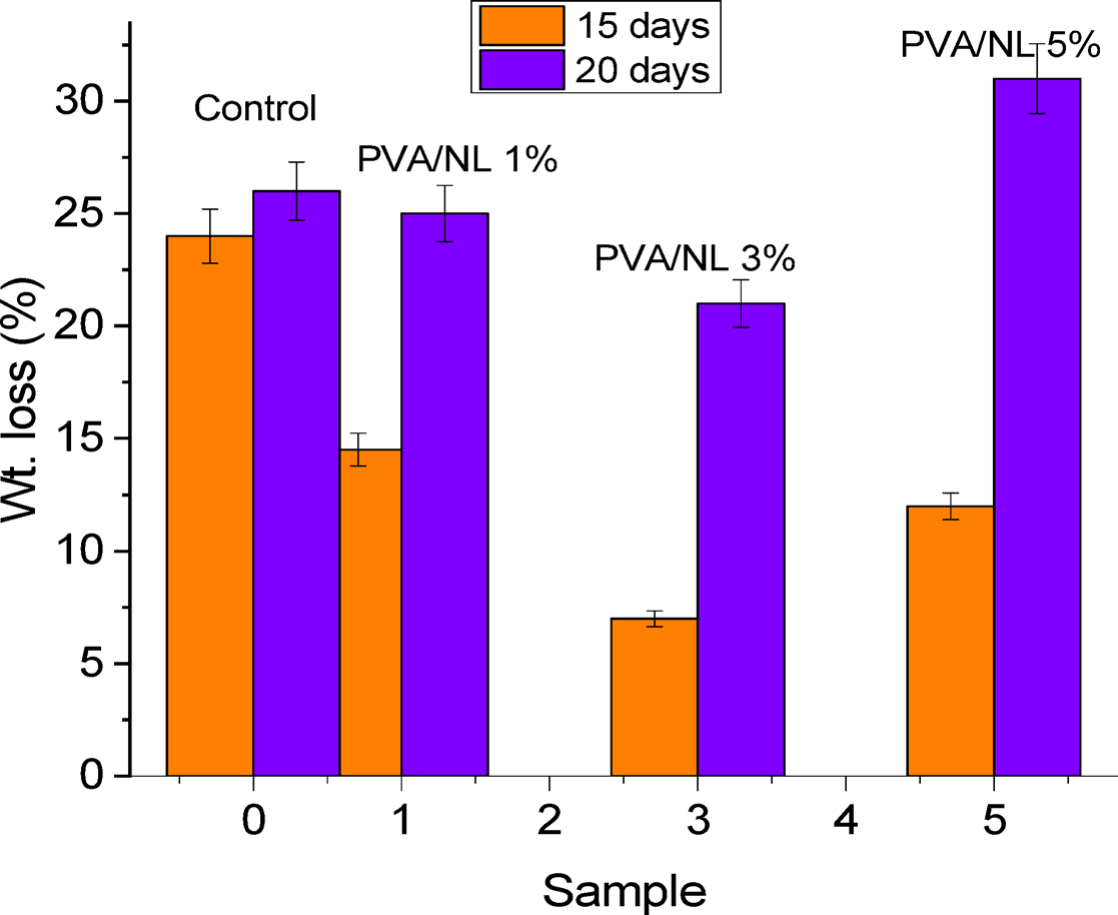
Wight loss of plum fruits coated with different PVA/NL composite after 15 and 20 days.

**Fig. 16:**
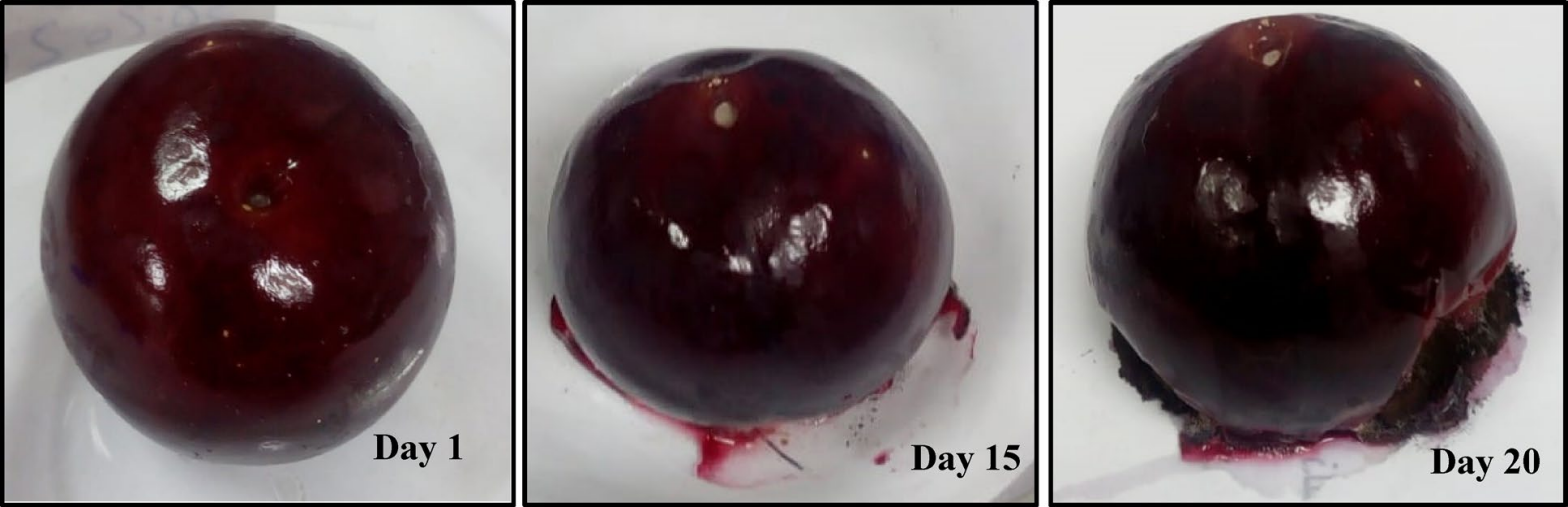
Preservation test for plum fruits coated with PVA /NL 5% for different time periods.

Although standard commercial plastic packaging materials such as polyethylene provide essential requirements for packaging, large volumes of fresh fruit are frequently wasted due to inadequate storage conditions^59^. Therefore, using the appropriate packaging material is crucial to maintain fresh fruits’ inherent quality, avoiding spoiling, and increasing shelf life. In this work, coating solutions from samples PVA/NL containing 1, 3, and 5% filler are prepared. For comparison, uncoated plums are stored under the same conditions and the examined fruits were left for 20 days. The fruits were observed with respect to freshness and weight loss. (Fig. 15) Generally, all showed minor changes in the first week. Whereas, sample fruit coated with PVA/NL 3% depicted the lowest value of weight loss after 15 days of storage (6.5%), then sample fruit coated with PVA/5% with value of 12.5%. After 20 days, the weight loss values were PVA/NL 3% < PVA/NL 5% < PVA/NL1% < control. These results can be explained in the light of gas permeability properties^60^. Nanocomposite films PVA/NL 1% and PVA/NL 3% demonstrated the lowest gas permeability value. However, the thickness of the nanocomposite film on the fruit after dipping is smaller than the free-standing film, so sample PVA/NL 3% showed higher preservation ability since it contains higher concentration of NL. Interestingly, sample fruit coated with PVA/NL 5% revealed total decay and deterioration after 20 days and leakage of fruit juice started at day 15. (Fig. 16) This behavior can be attributed to the high gas permeability of the nanocomposite that causes internal condensation on fresh food surfaces during aspiration, which can foster the growth of mold^61^. Moreover, NL is a natural material that can undergo biodegradation under the action of numerous extracellular enzymes, including quinine oxidoreductase, laccase, cellobiose, manganese peroxidase, and lignin peroxidase^62^. Thus, the presence of a high concentration of NL enhanced the deterioration of the surface and weight loss.

## Conclusion

Nanocomposite films of PVA containing different ratios of NA were prepared using a simple casting method. The films were characterized by FTIR, XRD, and water retention and their morphology was examined using SEM. The mechanical properties of the nanocomposite films showed clear plasticizing character by increasing the amount of NL. Investigation of UV transmission ability revealed the enhanced ability of the films to absorb or scatter UV light. Studying the optical properties of the nanocomposite films showed how varying quantities of nanolignin can tune the optical band gap of polyvinyl alcohol (PVA). These alterations could be advantageous for applications that require particular optical features. Moreover, the films demonstrated a decrease in the gas barrier ability for films containing 0.5 and 1% of NL. Increasing amounts of the incorporated filler led to deterioration of the gas barrier property. All films revealed antimicrobial and antioxidant activities. The ability of the dipping solution to preserve freshness and reduce weight loss of plum fruits was studied. The results showed that PVA/NL 3% could preserve the coated fruit for 20 days, whereas increasing the amount of NL to 5% in the dipping formulations boosted mold growth and fruit spoilage after 15 days. The results of this study confirmed the versatile properties of the prepared nanocomposite films, which enable their utilization in various applications, including active packaging material.

## Data Availability

All data generated or analyzed during this study are included in this published article.
